# Supplementary material for: Adult Hepatitis B Virus Vaccination Coverage in China from 2011 to 2021: A Systematic Review
Source: Vaccines (Basel). 2022 Jun 6;10(6):900. doi: 10.3390/vaccines10060900 (PMC9227759; doi:10.3390/vaccines10060900)
Supplement: Supplementary file 1 [file vaccines-10-00900-s001.zip › vaccines-1689186-supplementary.pdf]

Supplementary materials

Table S1. Detailed search strategies for PubMed.
Table S2. The AHRQ forms and modified version.
Table S3. Quality assessment of eligible studies

Table S1. Detailed search strategies for PubMed

#	Searches
1	Hepatitis B [MeSH]
2	hepatitis B[Title/Abstract]
3	#1 OR #2
4	Vaccines [MeSH]
5	Vaccination [MeSH]
6	vaccin*[Title/Abstract] OR immuni*[Title/Abstract]
7	#4 OR #5 OR #6
8	"Humans"[MeSH]
9	coverage [Title/Abstract] OR uptake [Title/Abstract] OR prevalence [Title/Abstract] OR proportion [Title/Abstract] OR status [Title/Abstract]
10	China [MeSH]
11	China [Title/Abstract] Or Chinese [Title/Abstract]
12	#10 OR #11
13	2011/01/01:2021/10/01[Date - Publication]
14	#3 AND #7 AND #8 AND #9 AND #12 AND #13

Table S2. The AHRQ forms and modified version.

Number	AHRQ Item	Modified AHRQ Item
Q1	Define the source of information (survey, record review)	Is the study defined the source of information (survey, record review)?
Q2	List inclusion and exclusion criteria for exposed and unexposed subjects (cases and controls) or refer to previous publications	Is the included population representative of the target population?
Q3	Indicate time period used for identifying patients	Is the included population representative of the national population?
Q4	Indicate whether or not subjects were consecutive if not population-based	Is the study list the inclusion and exclusion criteria for subjects or refer to previous publications?
Q5	Indicate if evaluators of subjective components of the study were masked to other aspects of the status of the participants	Is the method of data collection properly described?
Q6	Describe any assessments undertaken for quality assurance purposes (e.g., test/retest of primary outcome measurements)	Is the study indicated the time period used for identifying subjects?
Q7	Explain any patient exclusions from the analysis	Is the study described how missing data were handled?
Q8	Describe how confounding was assessed and/or controlled.	Is there a clear definition of hepatitis B vaccination status?
Q9	If applicable, explain how missing data were handled in the analysis	Is the study described any assessments undertaken for quality assurance purposes?
Q10	Summarize patient response rates and completeness of data collection	Summarize subjects' response rates and completeness of data collection
Q11	Clarify what follow-up, if any, was expected and the percentage of patients for which incomplete data or follow-up was obtained	

Table S3. Quality assessment of eligible studies.

Author, Year	Q1	Q2	Q3	Q4	Q5	Q6	Q7	Q8	Q9	Q10	Score
Wang SQ, 2011	Y	N	N	Y	N	Y	N	N	N	Y	4
Zhang XS, 2011	Y	Y	N	Y	Y	N	N	N	N	N	4
Zhang W, 2011	Y	Y	N	Y	Y	N	N	Y	N	Y	6
Chen YX, 2012	Y	N	N	Y	Y	Y	N	N	N	N	4
OuYang L, 2013	Y	Y	N	Y	N	Y	N	N	N	Y	5
Chen XQ, 2015	Y	Y	N	Y	Y	Y	N	N	N	Y	6
Yan H, 2015	Y	Y	N	Y	Y	Y	N	N	N	Y	6
Li ze, 2015	Y	N	N	N	N	N	N	N	N	N	1
Su YP, 2016	Y	Y	N	Y	Y	N	N	N	Y	N	5
Gao P, 2016	Y	Y	N	Y	Y	Y	N	N	N	N	5
Liu TT, 2017	Y	Y	N	Y	Y	Y	N	N	Y	N	6
Liang ZS, 2017	Y	N	N	N	Y	Y	N	N	N	Y	4
Ye X, 2017	Y	Y	N	Y	Y	Y	N	N	N	Y	6
Mai W, 2018	Y	Y	N	Y	N	Y	N	N	N	Y	5
Ma TL, 2019	Y	Y	N	Y	Y	N	N	N	Y	N	5
Yang K, 2019	Y	Y	N	Y	Y	Y	N	N	N	Y	6
Shi W, 2020	Y	Y	N	Y	Y	Y	N	N	N	N	5
Wan YM, 2020	Y	Y	N	Y	Y	N	N	Y	N	Y	6
Xu C, 2020	Y	Y	N	Y	Y	N	N	Y	N	Y	6
Xu Y, 2020	Y	Y	N	Y	Y	Y	N	N	N	Y	6
Yan L, 2021	Y	Y	N	Y	Y	Y	N	Y	N	N	6

Y: Yes; N: No; UC: Unclear.
